# Stratified shared genetic architecture of IBD and RA: an integrated analysis from polygenic overlap to directional heterogeneity

**DOI:** 10.3389/fimmu.2025.1711302

**Published:** 2025-12-05

**Authors:** Yiwen Jia, Yuntian Xia, Siyuan Chen, Guangming Feng

**Affiliations:** 1 Department of Gastroenterology, The Third Affiliated Hospital of Anhui Medical University, Hefei, China; 2 International Institute of Finance, School of Management, University of Science and Technology of China, Hefei, China

**Keywords:** MiXeR, FUMA, GWAS, shared genetic architecture, rheumatoid arthritis (RA), inflammatory bowel disease (IBD)

## Abstract

**Background:**

Inflammatory bowel disease (IBD) and rheumatoid arthritis (RA) are chronic immune-mediated disorders with overlapping clinical and immunological features, yet genome-wide genetic correlation (rg) between them has remained modest.

**Methods:**

We integrated multiple complementary approaches to dissect their genetic relationship, including MiXeR, LAVA, conditional/conjunctional FDR (cond/conjFDR), PLACO, and FUMA, using large-scale GWAS datasets of European ancestry.

**Results:**

MiXeR revealed extensive polygenic overlap between IBD and RA (Dice coefficient ≈ 0.46), encompassing hundreds of shared causal variants, while the global rg remained low (≈0.06). The weakened rg was attributable to directional heterogeneity, as many shared variants exhibited opposite effect directions across the two diseases. LAVA identified specific loci with significant positive local correlations, such as *TNFAIP3* (6q23), *COG6/TNFSF11* (13q14), and *JAK2* (7q36). Cond/conjFDR and PLACO uncovered thousands of pleiotropic SNPs, with high consistency across methods, confirming extensive genetic sharing. FUMA functional annotation highlighted enrichment of shared genes in T-cell activation, cytokine signaling, and Th1/Th17 differentiation pathways, with tissue enrichment observed in blood, spleen, intestine, and lung.

**Conclusions:**

Despite modest genome-wide correlation, IBD and RA share a high degree of polygenic risk, and the apparent paradox is explained by mixed effect directions of shared variants. These results provide a stratified view of their shared genetic architecture and offer new insights into common immunological pathways contributing to comorbidity. As the GWAS datasets were predominantly of European ancestry, the generalizability of these findings to non-European populations remains uncertain, validation in ancestrally diverse cohorts is needed.

## Introduction

Inflammatory bowel disease (IBD)—encompassing its two principal clinical subtypes, Crohn’s disease (CD) and ulcerative colitis (UC) (1)—and rheumatoid arthritis (RA) (2) are immune-mediated chronic inflammatory disorders. Clinically, these conditions can co-occur within individuals and families, show alternating courses over time, and display overlapping immunologic phenotypes (3, 4). Biologically, they share multiple immune pathways (e.g., antigen presentation, T-cell activation, and proinflammatory cytokine networks) within a pathogenic framework shaped by environment–microbiome interactions (5–9).

Over the past decade, genome-wide association studies (GWAS) have identified numerous susceptibility loci for IBD and RA, offering important clues to disease mechanisms (10). To date, more than 200 risk loci have been reported for IBD (including CD and UC), and over 100 genetic signals have been confirmed for RA in European populations (11–13). Nevertheless, estimates of genome-wide genetic correlation (rg) between the diseases have generally been moderate to low, giving rise to an apparent paradox: clinical and immunologic evidence suggests similarity, yet rg remains modest (14). This pattern implies that cross-disease genetic sharing may be obscured by more complex directional structure—for example, antagonistic pleiotropy in which the same locus exerts opposite effects on the two diseases, a phenomenon that appears concentrated in the major histocompatibility complex/human leukocyte antigen (MHC/HLA) region (15). At the same time, non-MHC regions may harbor broader concordant sharing with individually small effects. Accurately characterizing this “strongly discordant plus weakly concordant” mosaic cannot rely solely on traditional rg or single-trait GWAS.

To address these challenges, we introduce several complementary analytical tools to characterize the genetic overlap between IBD and RA from multiple angles. First, we use MiXeR to quantitatively decompose the polygenic architectures of the two diseases (16). MiXeR is a bivariate causal mixture model that, without being constrained by the overall correlation, estimates the number and proportion of shared non-zero genetic effects between traits (17–20). In other words, even if the genome-wide rg between IBD and RA is small, MiXeR can detect substantial polygenic overlap and quantify both the total number of shared risk variants and the distribution of disease-specific effect sizes. Second, we apply the LAVA framework for local genetic correlation analysis, partitioning the genome into approximately independent linkage disequilibrium (LD) blocks and evaluating the local correlation between IBD and RA within each region (21). This approach identifies genomic segments that contribute disproportionately to cross-trait association and determines the directionality (positive or negative) of local correlation, thereby revealing heterogeneity that is masked by genome-wide averages. For example, if negative correlation signals are concentrated in the MHC while positive signals arise in other immune-gene regions, LAVA can differentiate them and clarify how distinct biological pathways differentially shape IBD–RA relatedness. Finally, we employ conditional and conjunctional false discovery rate methods (condFDR/conjFDR) to pinpoint specific cross-trait co-localized risk loci (21). By jointly leveraging association evidence from both traits, condFDR/conjFDR increases statistical power to detect shared genetic variants—even when a variant does not achieve genome-wide significance in either single-trait GWAS—and has proven effective for uncovering pleiotropic loci underlying comorbidity. In the context of RA and IBD, this strategy is expected to reveal novel shared associations and identify variants that contribute to both diseases.

Taken together, MiXeR provides an overall quantitative assessment of genetic overlap, LAVA elucidates the local structure and directional heterogeneity of that overlap, and condFDR/conjFDR localizes concrete shared variants. The integrated use of these methods enables a comprehensive dissection of the magnitude and directionality of IBD–RA genetic sharing in European populations, offering multi-layered insights into their common genetic architecture and potential molecular mechanisms. By leveraging these advanced approaches, our study seeks to overcome the limitations of conventional analyses and to provide new perspectives on the genetic links and comorbidity mechanisms between IBD and RA.

## Methods

### GWAS sources for IBD and RA

Summary-level GWAS data for IBD and its subtypes (CD and UC) were obtained from the IIBDGC. The IIBDGC dataset were included in the IBD GWAS, from which 27,432 Europeans (6968 cases and 20,464 controls) and 20,883 Europeans (5956 cases and 14,927 controls) were included in the UC and CD GWAS, respectively. All of European ancestry, confirmed through radiologic, endoscopic, and histopathologic evaluations (22). The latest summary-level statistics for RA were obtained from the FinnGen GWAS results (https://r12.finngen.fi/), which comprised a total of 16314 patients with RA and 315115 controls. All the participants were of European ancestry (**Supplementary Table S26**). There is no overlap between these IBDs and RA data sources.

### MiXeR

MiXeR was applied to investigate the overall shared genetic architecture between IBD and RA (16). Univariate MiXeR analyses were first conducted to estimate each trait’s polygenicity and discoverability. Polygenicity was defined as the number of trait-influencing variants accounting for 90% of the heritability for each phenotype. Heritability was calculated as the cumulative effect of all trait-influencing variants, which are common variants exhibiting a non-zero additive effect on the trait. Discoverability was the average estimated effect size of causal variants.

Before performing bivariate models, we confirmed that the AIC and BIC values of the univariate models were positive, supporting sufficient power for bivariate MiXeR analyses. In the univariate MiXeR analysis, positive AIC values indicate that the GWAS summary statistics have sufficient power to justify the use of the MiXeR model compared to the LDSC model. In the bivariate MiXeR, a positive AIC value indicates adequate discrimination between the best model fit and the model assuming minimal or maximum possible polygenic overlap. Next, bivariate models were implemented to identify the number of unique and shared causal variants for each pair of traits. These models also provide estimates of the proportion of causal variants with concordant directions of effect. The Dice coefficient, an indicator of the proportion of polygenic overlap, is also computed.

MiXeR power analysis further: (1) Quantifies the proportion of phenotypic variance explained by causal SNPs reaching genome-wide significance at current sample sizes; (2) Estimates required sample sizes to explain larger proportions of SNP heritability, noting that traits with lower genetic discoverability demand larger samples.

Next, bivariate modeling approaches were applied to quantify the number of trait-specific and overlapping causal variants between each pair of phenotypes (23). These analyses also yielded estimates regarding the proportion of causal variants exhibiting concordant effect directions. Additionally, the Dice coefficient - a metric quantifying polygenic overlap - was calculated. The standard error of the Dice coefficient, estimated via parametric bootstrapping (1000 replicates) of z-scores in MiXeR (16). As a secondary analysis, we used MiXeR to examine the shared genetic architectures of CD and UC with RA. This secondary analysis shows how the shared genetic architecture of CD and UC compares with RA.

All MiXeR models used the 1000 Genomes Phase 3 European ancestry reference panel for LD information and only HapMap3 SNPs with MAF > 0.01 were included. We excluded variants located in extended long-range LD regions (including the extended the major histocompatibility complex (MHC) on chr6:25–35 Mb) to avoid bias from overly complex LD structure. The results are presented in a Venn diagram, illustrating the unique and shared polygenic components among traits.

Finally, LD score regression (LDSC) version 1.0.1 was employed to calculate heritability estimates and genetic correlations between trait pairs, quantifying the average degree of shared genetic effects (24). The analysis additionally computed standard errors for these parameters, representing the precision of the estimated genetic correlations and heritability values.

### LAVA

LAVA (Local Analysis of Variant Association) was an integrated framework for genetic correlations analysis (21). It can evaluate local heritability (h^2^_SNP_) and analyze conditional genetic relations between several phenotypes using partial correlation and multiple regression. LAVA was used to test the standard bivariate local rgs between IBD and RA. And the pairwise local rg tests on 2,495 genomic loci (the entire genome, p < 0.05/2495) applying multivariate genetic association analysis can provide more complex and conditional genetic relationships. The 1000 Genomes Project (phase 3, European ancestry) was used as the reference panel for LD estimation (25). The significance of local rg was adjusted using FDR (Benjamini–Hochberg) correction.

### Cond/conjFDR analyses

Cond/conjFDR analyses were employed to enhance the identification of loci related to each trait and to detect loci shared between IBD and RA. The condFDR method, built upon an empirical Bayesian statistical framework, utilizes the combined power of 2 GWAS data to improve the identification of genomic variants (26). This approach refines the selection of SNPs by considering their associations with the primary and secondary traits, identifying variants that are more likely to represent true associations despite not meeting genome-wide significance thresholds. For each trait pair, condFDR re-ranks the test statistics of the primary trait through adjusting for the SNP associations with the secondary trait. The cross-trait enrichment was assessed by constructing conditional quantile–quantile plots. To visually assess cross-trait enrichment patterns, conditional Q-Q plots were generated. These plots illustrate how the distribution of P-values for a primary trait varies according to its association strength with a secondary trait, stratified across three significance thresholds (P ≤ 0.1, 0.01, and 0.001). Cross-trait enrichment was demonstrated by leftward deflection from the expected line as the strength of association in the secondary trait increased, reflecting enrichment of shared genetic loci between IBD and RA. The conjFDR approach, an extension of condFDR, involves performing 2 condFDR analyses. The conjFDR value is defined as the maximum of the 2 corresponding condFDR values, providing a conservative estimate of the FDR for loci associated with both traits. Consistent with previous studies (20, 27–29), the significance levels for condFDR and conjFDR analyses were established at 0.01 and 0.05, respectively. To formally assess whether the observed effect directions between IBD (and subtypes) and RA differed from random expectation, we performed two-sided exact binomial sign tests on all SNPs identified through condFDR/conjFDR analyses. The null hypothesis assumed a 50% concordance rate. Statistical significance was evaluated using the binomial test implemented.

### PLACO

To enhance the credibility of the results, we employed the Pleiotropy Analysis using Combined Omics (PLACO) method to validate the findings obtained previously (30). PLACO is adept at identifying potentially pleiotropic SNPs between two traits. It operates by considering the composite null hypothesis, which posits that a variant is either not associated with any of the traits or is associated with just one of them. This approach is specifically designed to pinpoint pleiotropic loci between two traits. To ensure the reliability of our findings and to avoid the influence of variants with extremely large effect sizes that could produce spurious signals, we excluded variants with a Z‐score squared (Z^2^) greater than 80 (31). We considered SNPs with a PLACO p‐value less than 5 × 10−8 as significant pleiotropic variants.

### FUMA

We selected SNPs identified as significant by conjFDR as lead SNPs and input these lead SNPs into FUMA (functional mapping and annotation) v1.5.2 for functional mapping and annotation to identify linkage disequilibrium (LD)-independent genomic loci (31). We defined independent significant SNPs as those that reached a condFDR < 0.01 or conjFDR < 0.05 in their respective analyses and were in LD with each other at r² < 0.6. Using the 1000 Genomes Project reference panel of European ancestry, lead SNPs were defined as SNPs selected from the independent significant SNPs that were in LD with each other at r² < 0.1. If the distance between two loci was less than 250 kb, they were merged into a single locus (32). The novelty of lead SNPs was determined by examining whether the variants reached genome-wide significance (p < 5×10^-8^) in the summary statistics of both IBD and RA. Gene annotation for lead SNPs was based on their location within a gene or their distance to the nearest gene transcription start site. Relevant VEP annotations, CADD scores, and information on the nearest transcription start site were obtained from OpenTargets (https://genetics.opentargets.org/, v22.10). The genomic localization of lead SNPs was further verified using dbGaP (https://www.ncbi.nlm.nih.gov/gap/). Subsequently, gene expression, tissue-specific enrichment, and gene set enrichment analyses were performed in FUMA using genes associated with the lead SNPs. Gene expression analysis employed the transcripts per million (TPM) normalization method, which is robust to differences in library size and sequencing depth across samples. All analyses were corrected for multiple comparisons using FDR (Benjamini–Hochberg) adjustment.

## Results

### MiXeR

**Figure 1:**
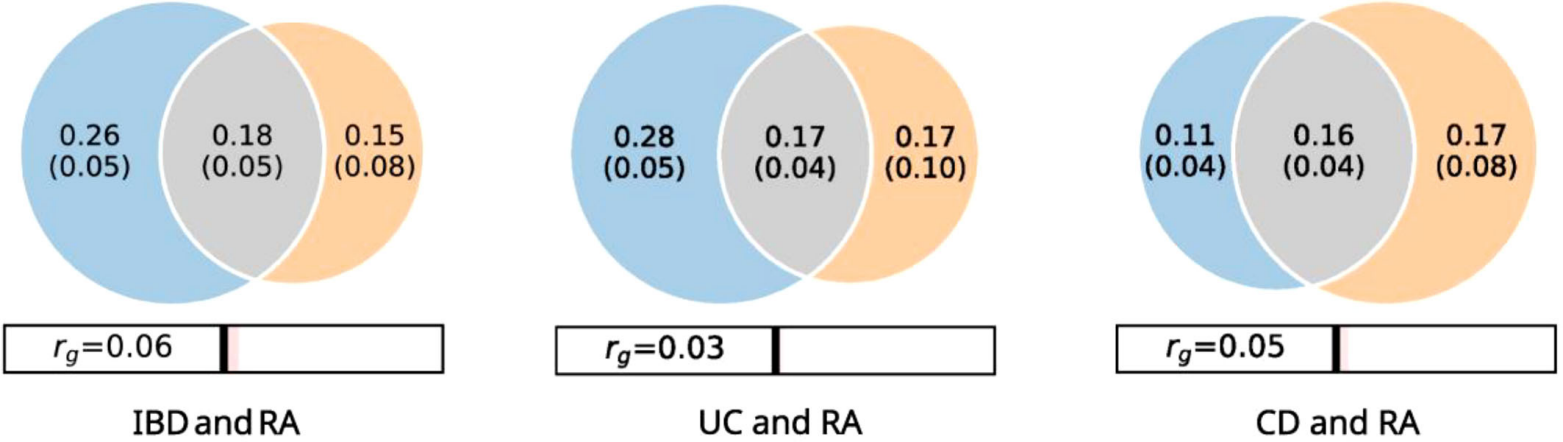
MiXeR Venn diagrams showing the estimated number of shared causal variants and genetic correlations (rg) of IBD and UC with each other and psychiatric disorders. The numbers of shared and trait-specific variants are shown in thousands with standard errors. The size of the circles reflects the degree of polygenicity.

The bivariate MiXeR analysis revealed a moderate polygenic overlap between UC and rheumatoid arthritis (RA). The Dice coefficient was approximately 0.43, indicating that roughly 43% of the causal variants for UC and RA are shared. MiXeR estimated on the order of a few hundred trait-influencing variants for each disease (UC = 0.28k, RA = 0.16k), of which roughly 0.17k potential causal variants were overlapping in this model. This overlap is reflected in a significant positive genetic correlation (global rg = 0.03, SE = 0.0134), suggesting that risk alleles in UC tend to confer risk in RA as well. Indeed, MiXeR’s shared-component analysis indicated just over half (52.6%) of the shared variants have concordant effect directions between UC and RA, slightly more than expected by chance (**Figure 1**, **Supplementary Table S1**). The genetic overlap between CD and RA appears slightly stronger in magnitude than that observed for UC. MiXeR analysis estimated a Dice coefficient about 0.53 for CD and RA, suggesting that over half of the causal variants for CD are shared with RA. In absolute terms, the model inferred roughly 0.11k causal variants underlying CD and 0.17k for RA, of which on the order of 0.16k variants were shared by both. Consistent with this, the genome-wide genetic correlation between CD and RA was significantly positive (rg = 0.05, SE = 0.0136). This correlation was higher than that for UC–RA. Thus, there is robust evidence that CD and RA have a common genetic basis to a considerable extent. The fraction of concordant effect alleles among shared variants was 53% in the MiXeR model, only slightly above 50% (**Figure 1**, **Supplementary Table S2**). When considering IBD as a combined phenotype (including both CD and UC), the genetic correlation with RA becomes stronger, reflecting the aggregate contribution of both subtypes. We observed a significant overall genetic correlation between IBD and RA (rg< =0.06, SE = 0.0103). IBD shares more genetic liability with RA than either subtype alone. MiXeR cross-trait modeling estimated that IBD and RA share a substantial portion of their polygenic architecture: the Dice coefficient was 0.46, corresponding to roughly 0.18k overlapping causal variants out of a few hundred in each disease. It is notable that the estimated overlap fraction for IBD–RA (0.46) falls between that of CD–RA and UC–RA, which is expected since IBD combines the two sub-phenotypes. MiXeR reports 54.3% of shared IBD–RA variants with concordant effects (risk alleles acting in the same direction), slightly higher than the subtype-specific fractions. This suggests that, when pooled, the combined IBD captures more of the consistently shared risk alleles with RA (**Figure 1**; **Supplementary Table S3**).

### LAVA

**Figure 2:**
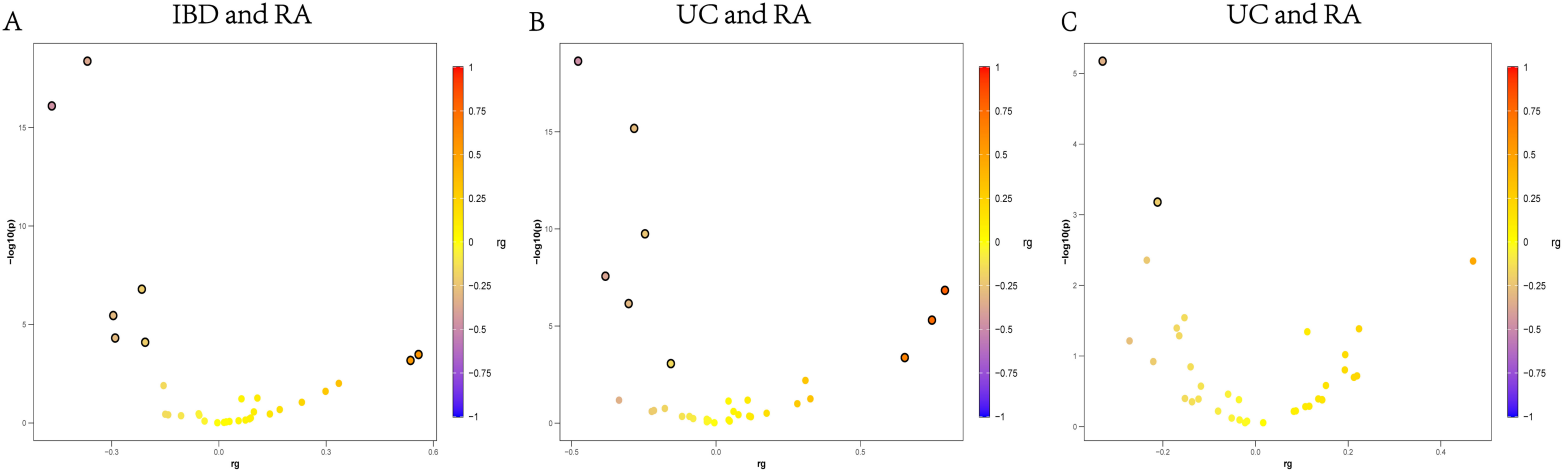
LAVA analysis of IBD and RA. The x-axis represents the local genetic correlation, and the y-axis represents the -log10 transformed LAVA values, with horizontal dashed lines reflecting significance (after Benjamini-Hochberg correction, with P < 0.05). **(A)** Local genetic correlation between IBD and RA. **(B)** Local genetic correlation between UC and RA. **(C)** Local genetic correlation between CD and RA. IBD, inflammatory bowel disease; CD, crohn’s disease; UC, ulcerative colitis; RA, rheumatoid arthritis.

Despite the modest global correlation, we identified three specific genomic regions with significant local genetic correlation between UC and RA (FDR < 0.05). These regions were located on chromosomes 6q, 13q, and 19q. Notably, the 6q23 locus (around 137–138 Mb on chr6) showed a high local genetic correlation (ρ = 0.79, 95% CI 0.52–1.00, p = 1.45×10^–7^, FDR = 4.6×10^–6^), indicating a strong sharing of genetic effects in this region. This locus coincides with the *TNFAIP3* gene region, an immune regulator known to be associated with autoimmunity. Similarly, a locus on chr13q14 (≈40.1 Mb) exhibited significant local correlation (ρ = 0.75, p = 5.0×10^–6^, FDR = 1.6×10^–4^). This region contains genes such as *COG6* and *TNFSF11*, which have been implicated in both RA and IBD. The third significant locus was on chr19q13 (≈44–45 Mb) with ρ ≈ 0.65 (p = 4.21×10^–4^, FDR = 1.35×10^–2^). These findings indicate that the UC–RA genetic overlap is not uniformly distributed across the genome but is driven in part by specific shared risk loci. We note that all three loci showed positive local correlations, implying that within these regions the alleles increasing UC risk also increase RA risk (consistent with concordant direction of effect) (**Figure 2A**; **Supplementary Table S4**). No significant negative local correlation signals were observed. In contrast to UC, the local genetic correlation analysis for CD and RA did not yield any genome-wide significant loci at FDR < 0.05. While several chromosomal regions showed nominal evidence of local ρ differing from zero, none survived multiple-testing correction in the CD–RA analysis (**Figure 2B**; **Supplementary Table S5**). The IBD vs RA local correlation analysis identified two genomic regions with significant local rg at FDR < 0.05. These correspond to loci on chromosome 7q and chromosome 13q, which were also implicated in the subtype analyses. The region on 7q36 (chr7: ~73–75 Mb) showed a significant positive local genetic correlation (ρ ≈ 0.54, 95% CI 0.24–0.90, p = 6.7×10^–4^, FDR = 2.1×10^–2^). Interestingly, this locus was not genome-wide significant in either UC–RA or CD–RA alone, suggesting that both subtypes contributed to a shared signal that only reaches significance when IBD is analyzed in aggregate. This 7q36 region includes genes such as *JAK2* and *SMURF2*, although further fine-mapping would be needed to pinpoint the causal gene. The second significant locus on chromosome 13q14 (around 40 Mb) had ρ ≈ 0.56 (CI 0.28–0.91, p = 3.3×10^–4^, FDR = 1.1×10^–2^). This is the same 13q locus highlighted in the UC–RA analysis, and its significance in IBD–RA confirms it as a robust shared region. As noted, this locus is near the *COG6* gene and the *TNFSF11* gene, among others, which have been associated with autoimmunity. It is worth mentioning that the 6q23 (*TNFAIP3*) locus, although a top signal in UC–RA, did not reach significance in the combined IBD–RA local analysis. Such an observation underscores the importance of considering disease subtypes: some genetic overlaps are specific to UC or CD and can be masked when only the aggregate is examined (**Figure 2C**; **Supplementary Table S6**).

### cond/conjFDR

**Figure 3:**
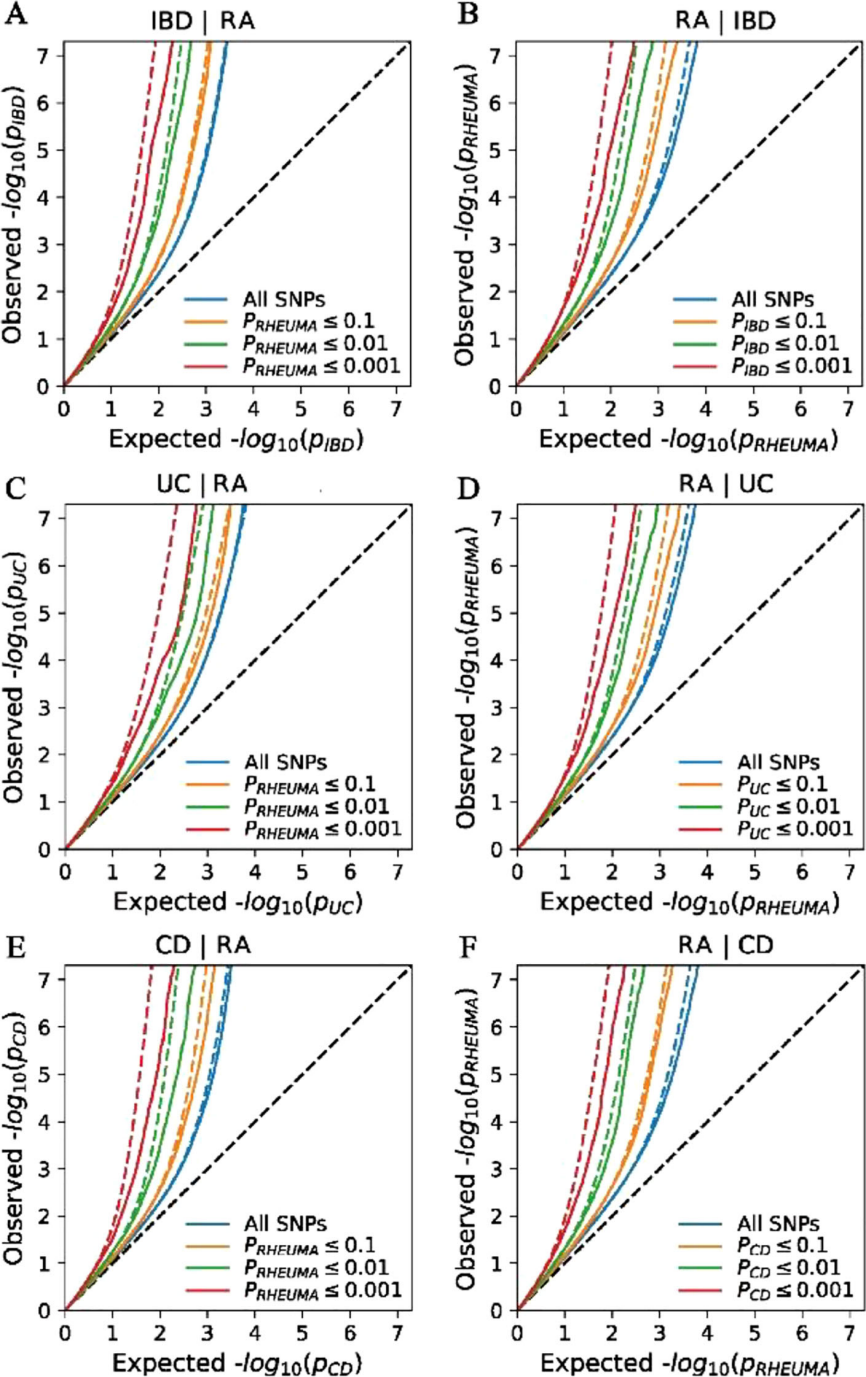
Conditional quantile-quantile plot. The dashed line indicates the expected line under the null hypothesis, and the deflection to the left indicates the degree of pleiotropic enrichment. **(A)** IBD-RA. **(B)** RA -IBD. **(C)** CD- RA. **(D)** RA -CD. **(E)** UC- RA. **(F)** RA -UC. RA, Rheumatoid arthritis; IBD, inflammatory bowel disease; CD, Crohn’s disease; UC, ulcerative colitis.

Using the conditional/conjunction FDR method, we uncovered numerous pleiotropic SNPs jointly associated with UC and RA. At a conjunction FDR < 0.05, 1,346 SNPs were identified as shared hits between UC and RA (**Figures 3C, D**; **Supplementary Figure S1**; **Supplementary Table S8**). The cond/conjFDR analysis identified a substantial set of overlapping SNP associations between CD and RA (**Figures 3E, F**; **Supplementary Table S7**). At conjFDR < 0.05, we found 1,280 SNPs jointly significant for CD and RA (**Supplementary Figure S2**). The conjunction FDR analysis combining all IBD revealed the largest set of shared variants with RA, as expected from the higher power of the combined phenotype. Using a significance threshold of conjFDR < 0.05, we identified 1,954 SNPs jointly associated with IBD and RA (**Supplementary Figure S3**). These nearly two thousand pleiotropic SNPs map to a wide array of loci, effectively covering most known IBD risk regions that also show association with RA (**Figures 3A, B**; **Supplementary Table S9**). A formal sign test confirmed that the observed effect direction concordance significantly deviated from random expectation (50%). Specifically, IBD–RA pairs showed 71.6% concordance (p < 2.2×10^-10^), CD–RA pairs 76.6% (p < 2.2×10^-16^), and UC–RA pairs 59.8% (p = 6.4×10^-13^). These results indicate that shared loci exhibit significant directional heterogeneity, with some variants showing opposite effects across diseases.

At the level of conjFDR<0.05, we found that most variations in IBD, UC, and CD did not reach significance in the original GWAS data (p<5e-8). Specifically, in IBD, UC, and CD, 51.4%, 55.9%, and 49.9% of SNPs did not reach significance, respectively. Among the shared loci, 1399 SNPs (71.6%) had consistent effect directions for IBD and RA and 555 (28.4%) had opposite effect directions (**Supplementary Table S7**). This supports the hypothesis that the lack of genetic correlation is due to mixed effect directions of variants shared by IBD and RA. The two most significant loci exerted opposite directions of effect for IBD and RA. The first was an ncRNA intronic variant (rs12212247) in the polymorphisms of the centrosomal gene, *CEP43* (also linked to numerous other traits, including lung cancer and stem cell myeloproliferative disorder) (33, 34). The most significant locus (rs12720356) with a concordant direction of effect was *TYK2*, which encodes tyrosine kinase 2 and has been linked to an array of multiple sclerosis, psoriasis and type 1 diabetes (35–37). As a verification, we compared the results obtained from LAVA and cond/conjFDR, and found that the directions of multiple variants remained consistent in both sets of results (**Supplementary Table S10**).

### PLACO

**Figure 4:**
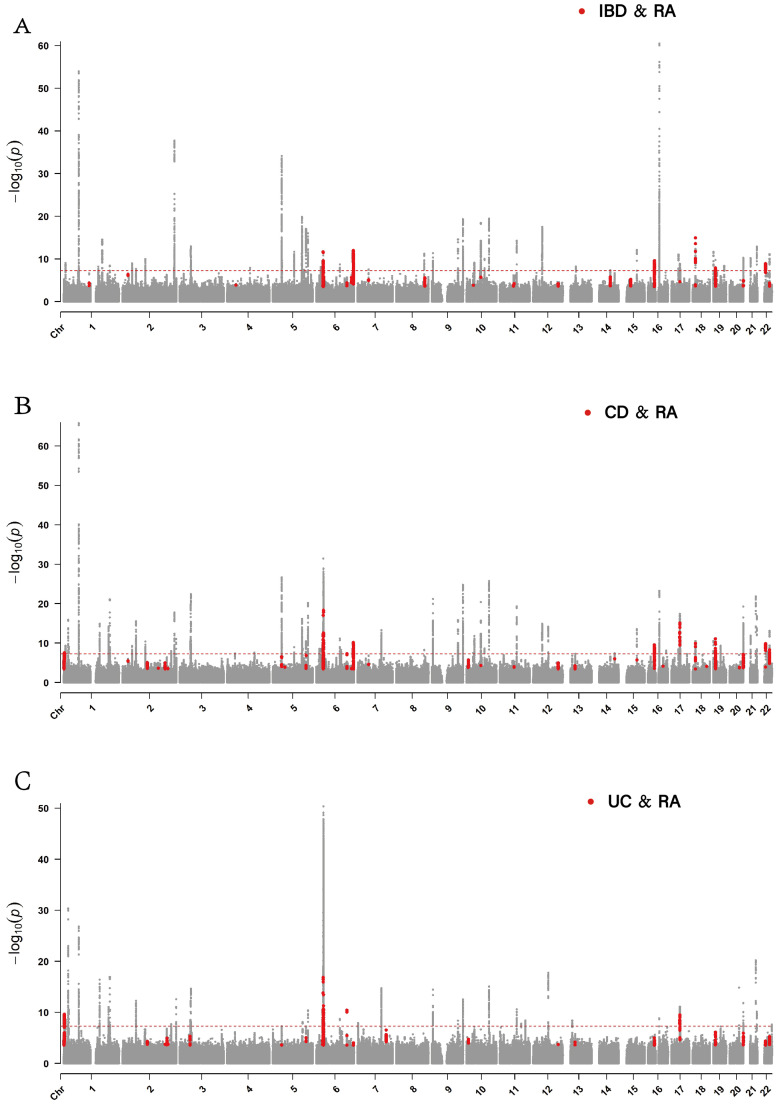
The Manhattan plot shows the SNP distribution of IBD and its subtypes, with the highlighted red areas indicating the overlap of PLACO and cond/conjFDR results. The x-axis stands for the chromosomalnumber and position and the y-axis represents -log10 transformed conjFdr values with a dotted horizontal line reflecting significance (p = 5e^-8^). **(A)** Manhattan plot of IBD and RA. **(B)**Manhattan plot of CD and RA. **(C)** Manhattan plot of UC and RA. RA, Rheumatoid arthritis; IBD, inflammatory bowel disease; CD, Crohn’s disease; UC, ulcerative colitis.

A PLACO pleiotropy analysis was conducted on three trait pairs exhibiting significant correlation. The results identified 7,064, 6,043, and 5,036 SNPs as pleiotropic loci between RA and IBD, CD, and UC, respectively (**Supplementary Table S11**-**13**). Notably, when comparing the cond/conjFDR results for IBD versus RA, we found that 1,421 SNPs were overlapping, and the effect direction of each SNP remained consistent (**Figure 4A**; **Supplementary Table S11**). When comparing the cond/conjFDR results for UC versus RA and CD versus RA, 1,160 and 719 SNPs, respectively (**Figures 4B, C**; **Supplementary Table S12**, **13**), were found to be identical. Among all results, only one SNP (rs2004640) was found to have a different effect direction in UC versus RA compared to the previous results (**Supplementary Table S13**). The PLACO results exhibited high consistency with the cond/conjFDR results. Among the three trait pairs, we observed a substantial number of overlapping non-significant SNPs below the threshold of 5e-8, which may contain novel SNPs with genetic correlations (**Figure 4**).

### FUMA

The variant effect predictor (VEP) analysis of the 128 lead SNPs in the loci shared between IBD and RA indicated that 46 were intronic, 58 intergenic, 1 downstream, 4 upstream. Eight of the 128 variants had Combined Annotation-Dependent Deletion (CADD) scores >12.37, indicating possible deleteriousness. The CADD value of the SNP rs2066845 is the highest (29.8) (**Supplementary Tables 14**). There are 109 and 66 lead SNPs for CD and UC, respectively (**Supplementary Tables 15**, **16**). For mapping genes, there are 372 for IBD, 278 for CD and 234 for UC (**Supplementary Tables 17**-**19**).

The GO annotation analyses were performed on the 260 mapped genes in IBD, and the results showed that in terms of biological processes, the top five aspects of gene enrichment were ‘T cell activation’, ‘regulation of T cell activation’, ‘regulation of leukocyte cell-cell adhesion’, ‘lymphocyte differentiation’ and ‘leukocyte differentiation’. In terms of cellular components, ‘sperm midpiece’, ‘side of membrane’, ‘pore complex’, ‘endosome lumen’ and ‘COP9 signalosome’ were the main directions of gene enrichment. In addition, genes were enriched for 12 molecular functions processes, including ‘protein tyrosine kinase activity’, ‘growth factor receptor binding’, ‘cytokine receptor binding’, ‘cytokine receptor activity’ and ‘cytokine binding’.

**Figure 5:**
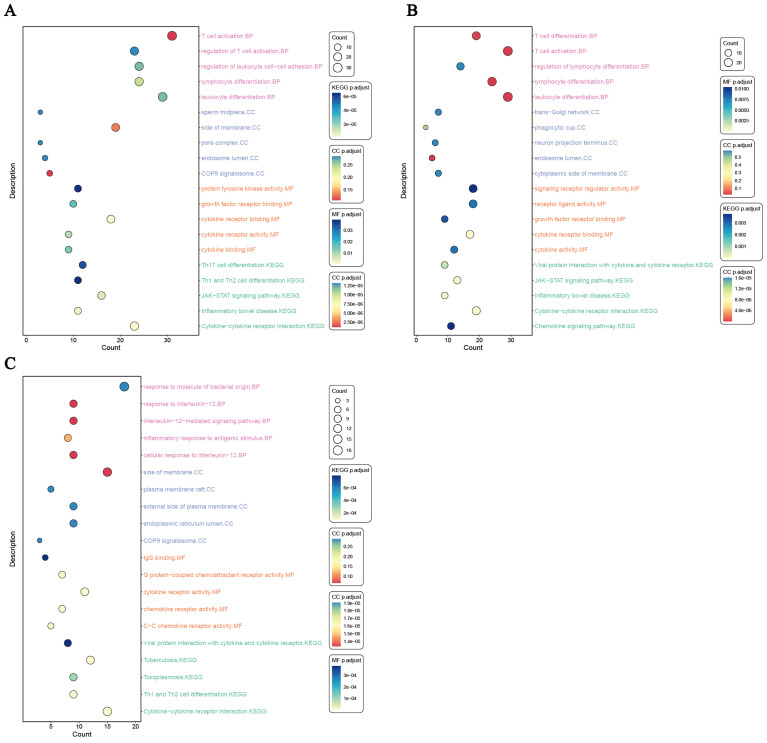
The results of enrichment of the shared genetic architecture of lead SNPs in distinct loci associated with IBD and RA (after Benjamini-Hochberg correction, with P < 0.05). **(A)** Enrichment analysis between IBD and RA. **(B)** Enrichment analysis between CD and RA. **(C)** Enrichment analysis between UC and RA. IBD, inflammatory bowel disease; CD, crohn’s disease; UC, ulcerative colitis; RA, rheumatoid arthritis.

KEGG pathway enrichment analysis identified 8 biological processes where shared genes were significantly enriched, with ‘Th17 cell differentiation’, ‘Th1 and Th2 cell differentiation’, ‘JAK-STAT signaling pathway’, ‘Inflammatory bowel disease’ and ‘Cytokine-cytokine receptor interaction’ being the top five processes (**Figure 5**; **Supplementary Table S19**). The results of the enrichment analysis for CD and UC can be seen in (**Figure 5**; **Supplementary Table S20**, **21**).

**Figure 6:**
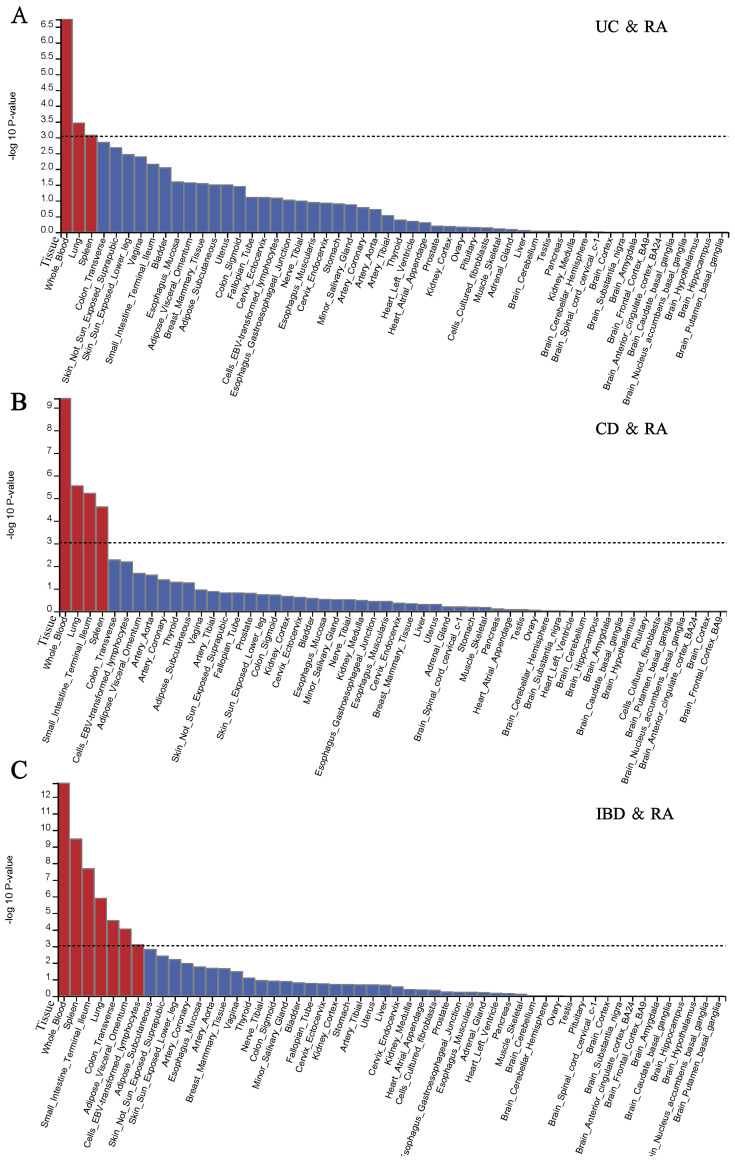
Gene expression in 54 GTEx tissue types for genes linked to lead SNPs in distinct loci significantly associated with IBD and RA. Significant enrichment (P<0.05 after Bonferroni correction, two-sided tests) is highlighted in red. **(A)** Tissues enrichment results between UC and RA. **(B)** Tissues enrichment results between CD and RA. **(C)** Tissues enrichment results between IBD and RA.

Analysis of Genotype-Tissue Expression (GTEx) samples showed that genes linked to the SNPs in IBD were significantly expressed in the blood, spleen, small intestine terminal ileum, lung, colon transverse, adipose visceral omentum and cells EBV-transformed lymphocytes. In UC, we found that the gene expression of the identified SNPs was mainly enriched in the blood, spleen, and lungs. As for the CD, tissue expression analysis shows that CD related gene expression mainly exists in the blood, lung, small intestine terminal ileum, spleen and cells EBV-transformed lymphocytes (**Figure 6**; **Supplementary Table S22**, **23**, **24**).

## Discussion

Our study used multiple methods to estimate the number and proportion of shared pathogenic variants between IBD and its subtypes and RA, and we found that at the genome-wide level, IBD and RA exhibit a low but significant overall genetic correlation (rg = 0.06, P < 0.001). To quantify the degree of polygenic overlap between IBD and RA at the genome-wide level, we fitted a MiXeR bivariate model to the GWAS summary statistics of both diseases. This result revealed extensive genetic overlap with heterogeneity of effects: many shared risk loci have discordant effect directions between IBD (and its subtypes) and RA, which weakens the overall rg. Next, we further pinpointed genetic overlap in specific regions using LAVA, estimating whether significant genetic overlap exists between IBD and RA at particular genomic regions. The results support the contribution of specific genomic regions to the shared susceptibility of IBD and RA. We further used cond/conjFDR to discover SNPs that are significantly associated with both traits, and validated the results with PLACO. Finally, we used FUMA to functionally annotate the significant SNPs across the different traits; these annotations help to elucidate the potential biological functions and related pathways of the identified shared loci.

Specifically, the proportion of polygenic risk shared between CD and RA is higher than that between UC and RA. This aligns with the differences in immune bias between the two IBD subtypes: CD tends toward a Th1-type immune response, whereas UC is more Th2-biased, and RA is typically Th1-driven (38). This result is also consistent with epidemiological data — RA has a higher comorbidity rate in CD patients, suggesting a closer potential genetic link between the two (39). We further utilized the cond/conjFDR method to mine common genetic risk loci between IBD and RA. The conditional Q-Q plot shows that when one disease is tested at a less stringent significance threshold, the statistical test for the other disease deviates significantly from the null expectation, demonstrating genetic enrichment and a shared genetic background between IBD and RA.

Delving deeper into specific shared genetic factors, all three analysis methods consistently indicated that IBD and RA have several common risk genes and loci. Using the conjFDR approach, we identified multiple risk loci that reached significance in both diseases, and these shared loci cover previously reported classic autoimmune-related genes. For example, the *TNFAIP3* gene (encoding the A20 protein, a negative regulator of the NF-κB pathway) is located on chromosome 6q23, and variants in this gene are implicated in multiple autoimmune diseases including RA and IBD (40). This locus exhibited associations in both RA and IBD in our analysis, reflecting a shared inflammatory signal regulation mechanism. Another example is the *IL23R* gene (chromosome 1p31), which mediates the Th17 immune axis; it was initially found to harbor a strongly associated variant in CD and has also been confirmed as a susceptibility factor for RA (41). The IL-23/IL-17 pathway plays an important role in both intestinal and joint inflammation, and the shared involvement of *IL23R* reinforces the immunological basis for the comorbidity of the two diseases (42). Similarly, variants in the *PTPN2* gene (encoding a non-receptor protein tyrosine phosphatase involved in T cell signaling) are associated with CD, UC, and RA, suggesting that this gene plays a coordinated role in autoimmune pathology across multiple organs. Additionally, integrating the local correlation and conjFDR results, we noted that gene regions such as *UBE2L3* showed significant associations in both IBD (especially CD) and RA, whereas IRF5 appeared in shared signals specific to UC (43). These pieces of evidence support that IBD and RA share a subset of key immune regulatory gene networks. However, it should be noted that each disease also has its own specific risk loci (for example, *NOD2* is mainly specific to CD, and *PTPN22* is mainly specific to RA), indicating that the common genetic basis accounts for only part of the risk, with the remaining risk coming from disease-specific genetic factors (44). This “commonality and difference” pattern implies that in terms of pathogenesis, IBD and RA have common immune pathways as well as distinct pathogenic mechanisms.

Our tissue expression analysis using FUMA, based on GTEx v8 data, showed that genes mapped to pleiotropic loci were significantly enriched in immune-related tissues. We observed strong expression enrichment in whole blood, spleen, lung, and small intestine–terminal ileum. These findings are consistent with the immune-mediated nature of both IBD and RA, and support the role of T-cell activation and cytokine signaling in their shared pathogenesis.

In our genetic analyses, we deliberately removed the highly polymorphic Major Histocompatibility Complex (MHC) region on chromosome 6 to prevent it from dominating the detection of shared signals. The MHC/HLA region is the strongest genetic association locus in many immune-related diseases, and RA and IBD are no exception (for example, in RA, the HLA-DRB1 “shared epitope” contributes significantly to risk; in IBD, certain HLA alleles have notable effects) (45, 46). In our analyses excluding MHC, we still detected a significant overall genetic correlation between IBD and RA and multiple shared loci, indicating that genes outside the HLA region (i.e., non-MHC genes) also play important roles in the co-morbidity of the two diseases. Overall, the strategy of excluding MHC improved the resolution of the genetic overlap between IBD and RA, ensuring that the results mainly reflect non-HLA genetic commonality rather than just the effect of a single MHC hotspot.

This study has several limitations. First, while we identified pleiotropic loci associated with both IBD and RA using multiple statistical methods, these loci have not yet been functionally or experimentally validated. The biological mechanisms underlying these associations remain to be elucidated. Future work should include experimental assays (e.g., reporter gene assays, CRISPR/Cas9 editing, or *in vitro* immune cell models) to verify the functional relevance of these shared variants. Secondly, our study’s data were limited to populations of European ancestry to ensure a consistent genetic structure. As sample sizes grow, it is critical to prioritize the inclusion of individuals of non-European ancestry. The potential consequences of excluding diverse ancestral groups include inequitable distribution of the benefits of genetic research and the exacerbation of existing health disparities (47–49). Therefore, as biobanks continue to expand, future studies should incorporate more diverse ancestral samples to validate these findings. This shift toward more diverse samples will enhance the generalizability of the results and improve our understanding of genetic variation across populations.

The observed stratified shared genetic architecture between IBD and RA, characterized by extensive polygenic overlap, directional heterogeneity, and enrichment of immune pathways, may inform several clinical translational directions. First, shared genetic components may help risk stratification, by indicating subsets of patients with one disease who could merit closer surveillance for the other (21, 50). Second, convergence on common immune pathways could help prioritize candidate biological targets with potential relevance across both conditions (51, 52). Third, recognition of mixed effect directions at shared loci cautions that genetically defined subgroups may respond differently, supporting the rationale for future genetically informed patient stratification in comorbid IBD and RA (53). These implications are hypothesis-generating and require validation in functional and clinical studies.

In summary, this study found that the reason for the lack of a stronger genetic correlation between IBD and RA is the presence of shared variants with opposite effect directions. Subsequent analyses clarified the overlapping genomic loci and identified those involved in effector functions and regulatory mechanisms. Together, these findings deepen our understanding of the shared polygenic genetic architecture between IBD (including its subtypes) and RA, further supporting the view that these two diseases have overlapping neurobiological mechanisms. A key limitation of this work is that all summary-level GWAS data analyzed were derived predominantly from individuals of European ancestry. Consequently, the generalizability of our findings to Asian and other non-European populations remains to be determined, underscoring an important knowledge gap and the need for replication and extension in diverse cohorts.

## Conclusions

Despite a modest genome-wide genetic correlation, IBD and RA share substantial polygenic risk with marked directional heterogeneity, helping to reconcile clinical overlap with low rg. Cross-trait discovery frameworks consistently uncovered large sets of pleiotropic variants, supporting extensive genetic sharing beyond genome-wide significance thresholds. Functional mapping indicated enrichment of shared genes in T-cell activation, cytokine signaling, and Th1/Th17 differentiation, with tissue enrichment in blood, spleen, intestine, and lung, pointing to common immunological pathways. Collectively, these results provide a stratified view of IBD–RA genetic architecture and a foundation for mechanistic and translational studies.

## Data Availability

The original contributions presented in the study are included in the article/**Supplementary Material**. Further inquiries can be directed to the corresponding author.
