# Supplementary figures and images for: Stratified shared genetic architecture of IBD and RA: an integrated analysis from polygenic overlap to directional heterogeneity
Source: Front Immunol. 2025 Dec 5;16:1711302. doi: 10.3389/fimmu.2025.1711302 (PMC12715422; doi:10.3389/fimmu.2025.1711302)

A

IBD and RA

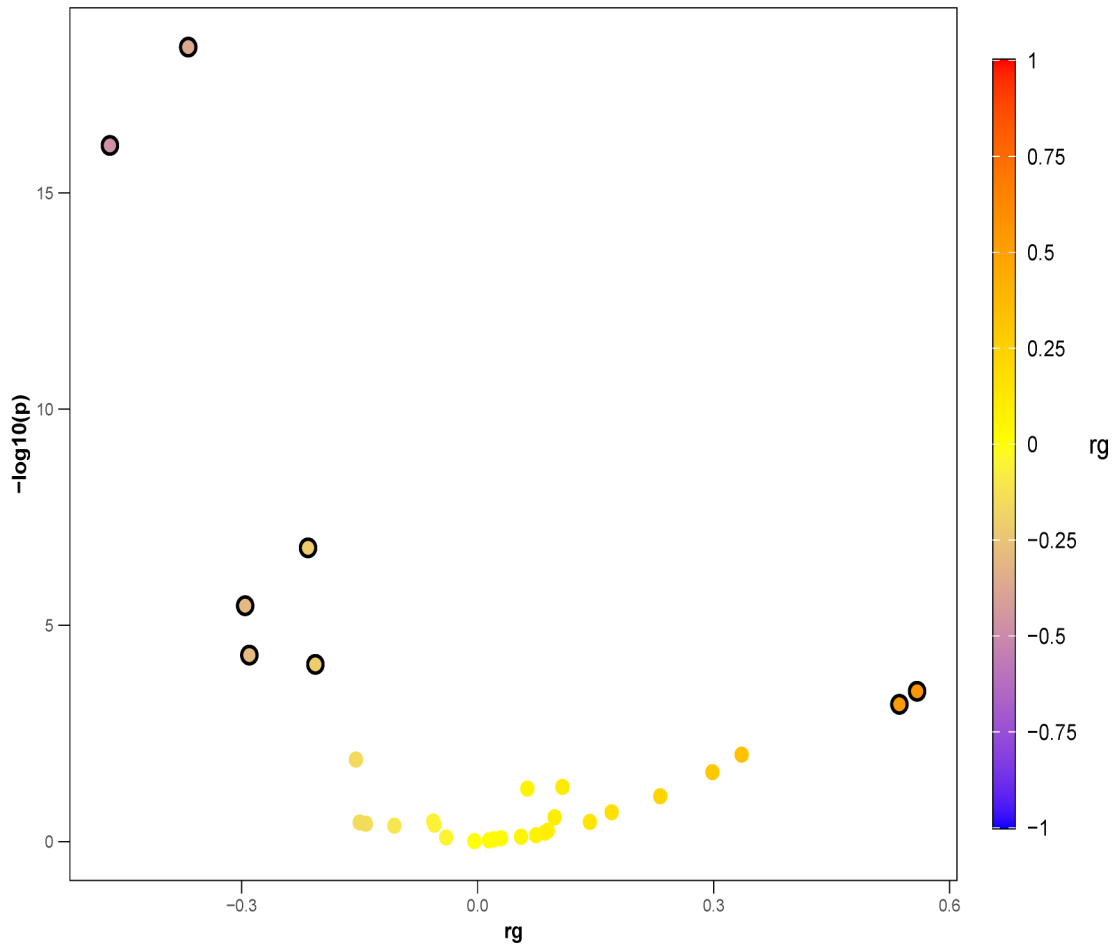

B

UC and RA

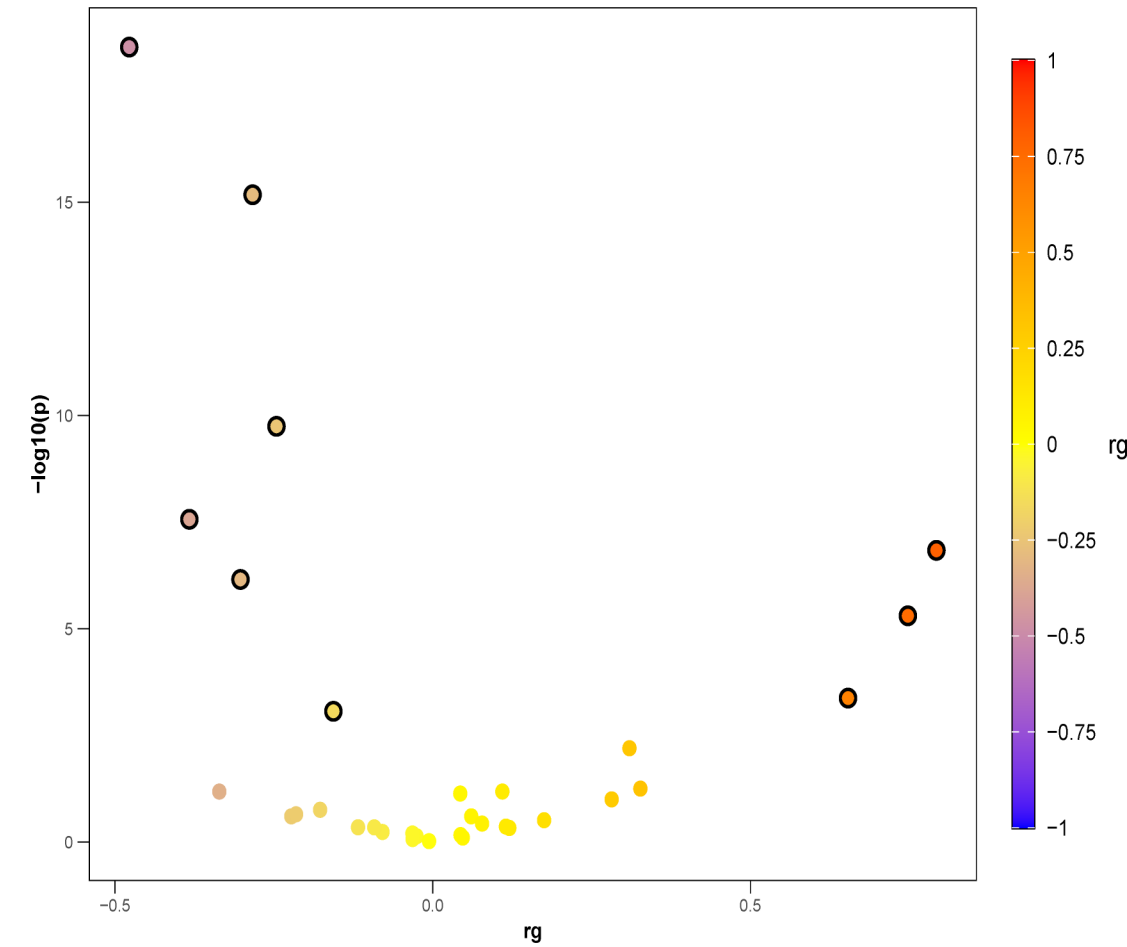

C

UC and RA

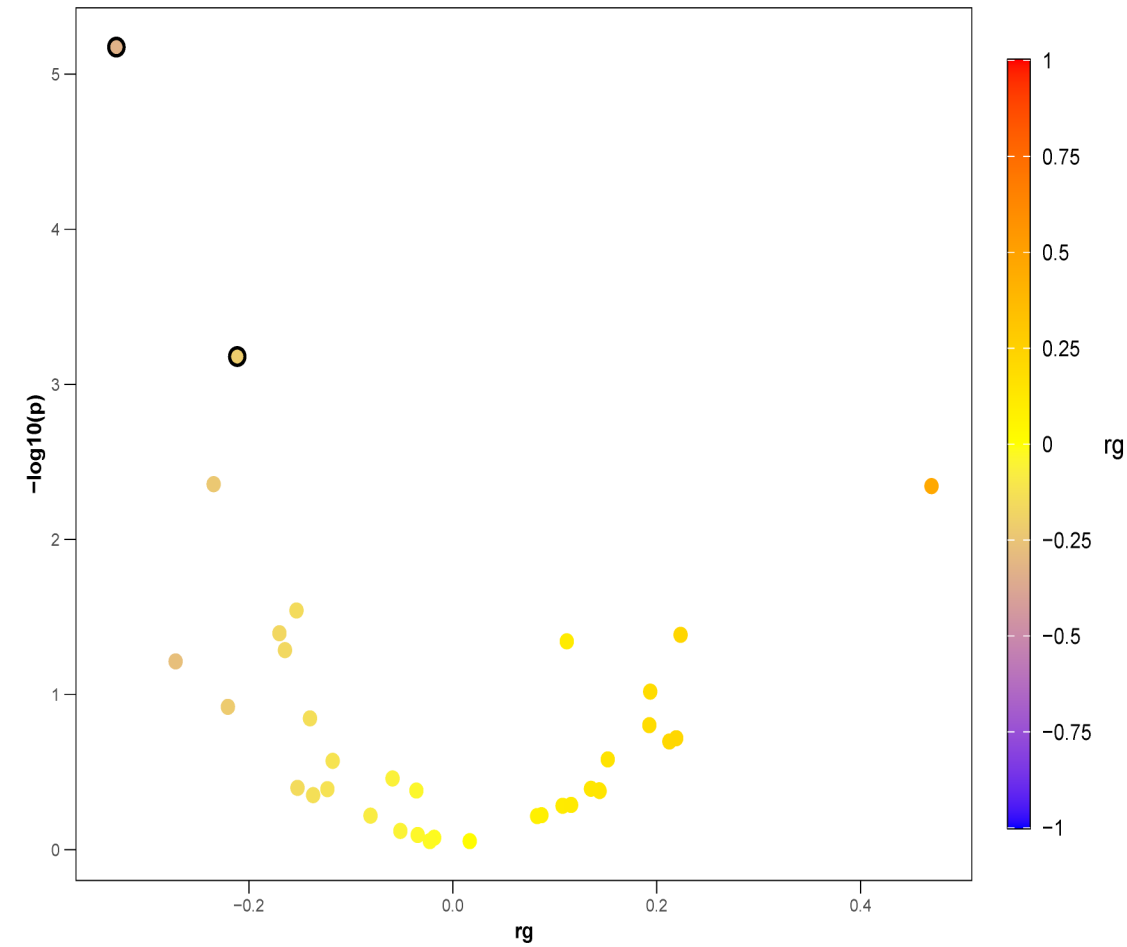

Supplement: Supplementary file 2 [file Image1.pdf]

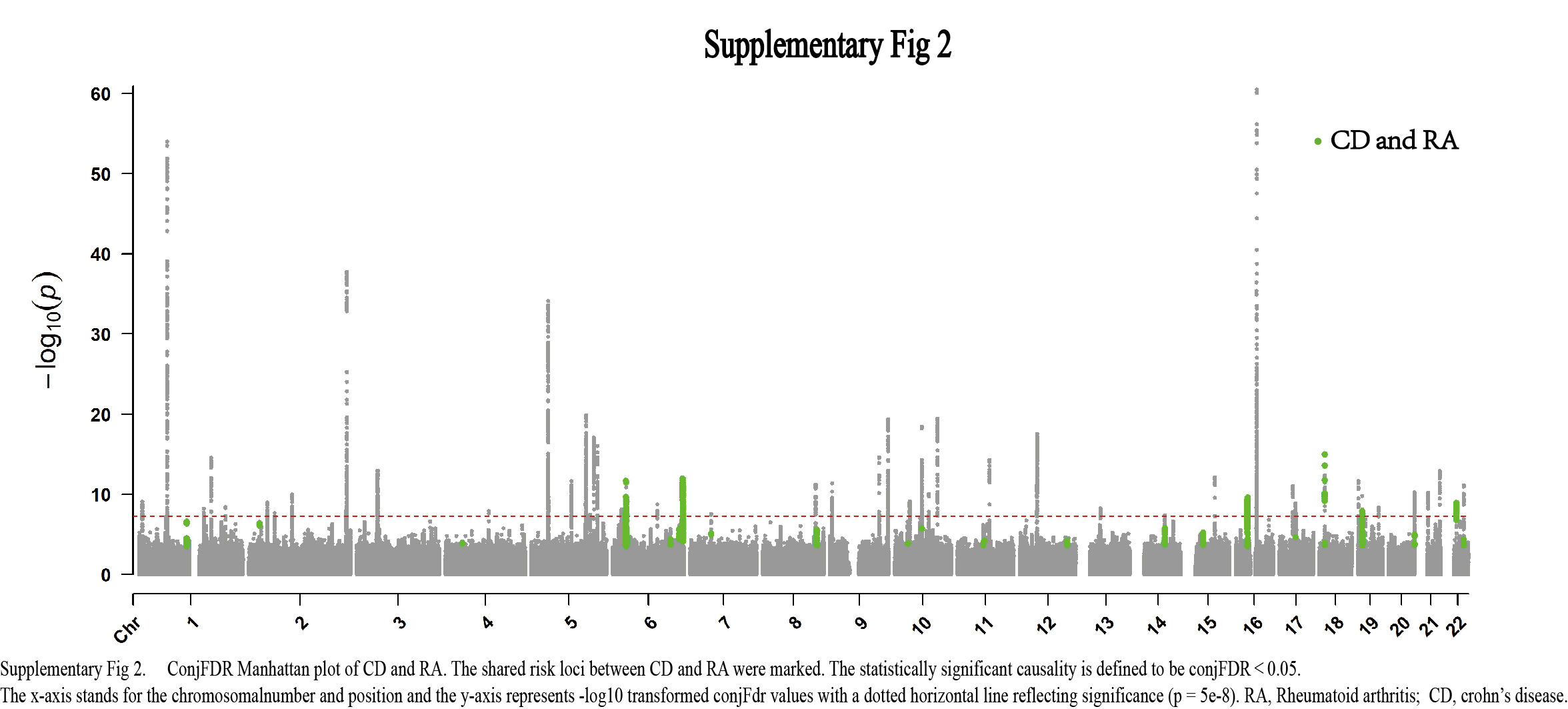

Supplement: Supplementary file 3 [file Image2.jpeg]

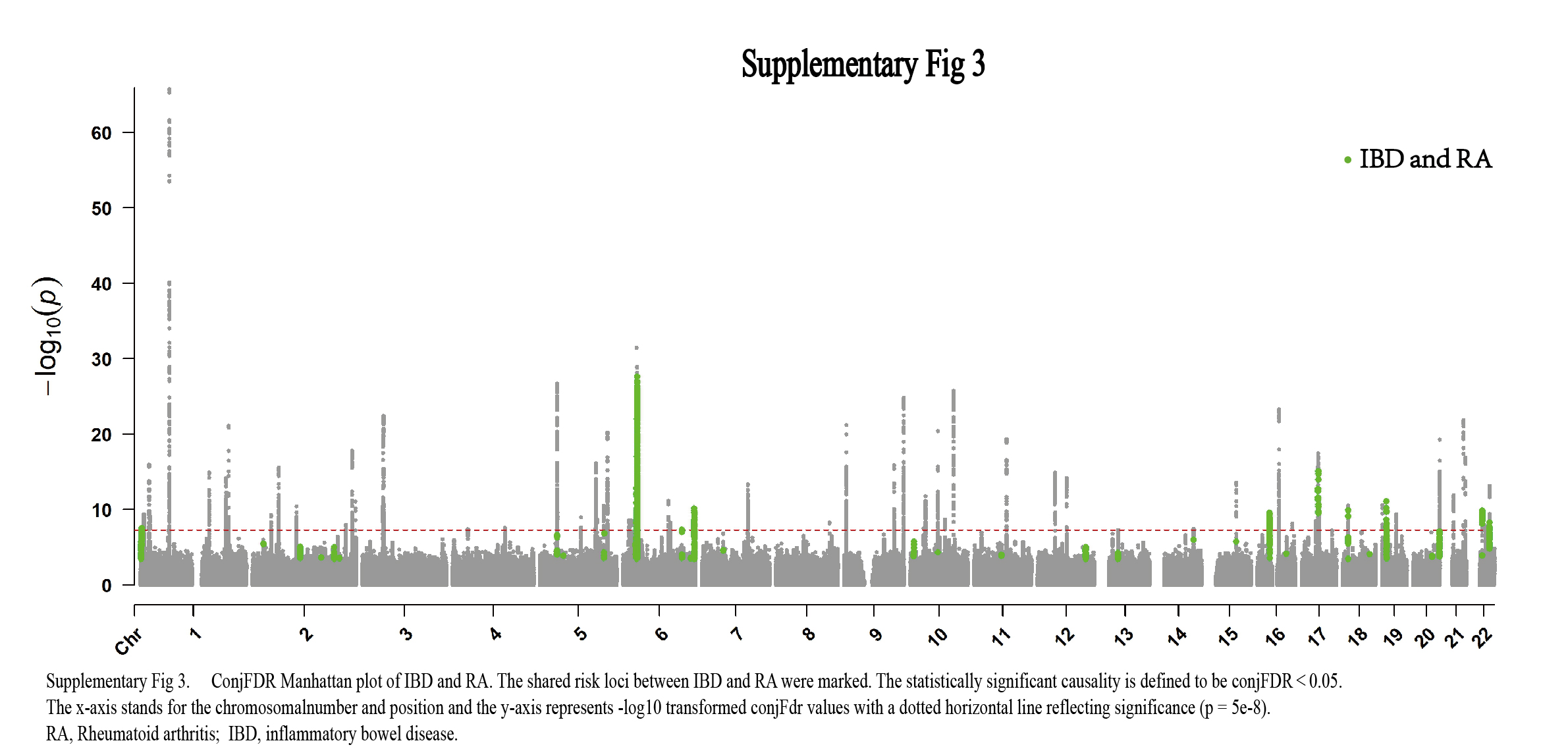

Supplement: Supplementary file 4 [file Image3.jpeg]
